# Quantum twin interferometers

**DOI:** 10.1126/sciadv.aea0816

**Published:** 2025-10-31

**Authors:** Wei Du, Shuhe Wu, Dong Zhang, Jun Chen, Yiquan Yang, Peiyu Yang, Jinxian Guo, Guzhi Bao, Weiping Zhang

**Affiliations:** ^1^ Tsung Dao Lee Institute and School of Physics and Astronomy, Shanghai Jiao Tong University, Shanghai 200240, China.; ^2^ Shanghai Research Center for Quantum Sciences, Shanghai 201315, China.; ^3^ Shanghai Branch, Hefei National Laboratory, Shanghai 201315, China.; ^4^ Collaborative Innovation Center of Extreme Optics, Shanxi University, Taiyuan, Shanxi 030006, China.

## Abstract

Quantum-correlated interferometer is an emerging tool in quantum technology that offers classical-limit-breaking phase sensitivity. However, to date, there exists a configurational bottleneck for its practicability due to the low phase-sensing power limited by the current detection strategies. Here, we establish an innovative development termed as “quantum twin interferometer” with dual pairs of entangled twin beams arranged in the parallel configuration, allowing full exploitation of the quantum resource through the configuration of entangled detection. We observe the distributed phase sensing with 3-decibel quantum noise reduction in phase-sensing power at the level of milliwatts, which advances the record of signal-to-noise ratio so far achieved in photon-correlated interferometers by three orders of magnitude. The developed techniques in this work can be used to revolutionize a diversity of quantum devices requiring phase measurement.

## Teaser

Quantum twin interferometer advances SNR in photon-correlated interferometers by three orders, revolutionizing phase measurement.

## INTRODUCTION

Over recent decades, interferometric techniques have evolved into indispensable phase measurement tools with broad applications spanning navigation systems (*1*, *2*), electromagnetic field sensing (*3*, *4*), and gravitational wave detection (*5*, *6*). The phase sensitivity of an interferometer is jointly limited by quantum noise fluctuations $\delta \hat{I}=\sqrt{\left\langle {\hat{I}}^{\;2}\;\right\rangle -{\left\langle \hat{I}\right\rangle }^{2}}$ and the slope of the interference fringes $|\;\partial_{\varphi }\left\langle \hat{I}\right\rangle \;|$ , which can be determined by

$$\delta \varphi =\frac{\delta \hat{I}}{|\;\;{\text{∂}}_{\varphi }\left\langle \hat{I}\right\rangle \;\;|}$$ (1)

Although linear interferometers demonstrate remarkable progress, their sensitivity remains constrained by the standard quantum limit (SQL), i.e., $\delta \varphi_{l}\propto 1∕\sqrt{N}$ , where N represents the mean photon number during phase interrogation (*7*). Breakthroughs in nonlinear optics enables the generation of nonclassical states with quantum correlation and manipulation of the quantum statistics of photons (*8*–*11*), leading to the advanced interferometry for detecting signals immersed in vacuum fluctuation (*12*–*14*).

**Fig. 1.**
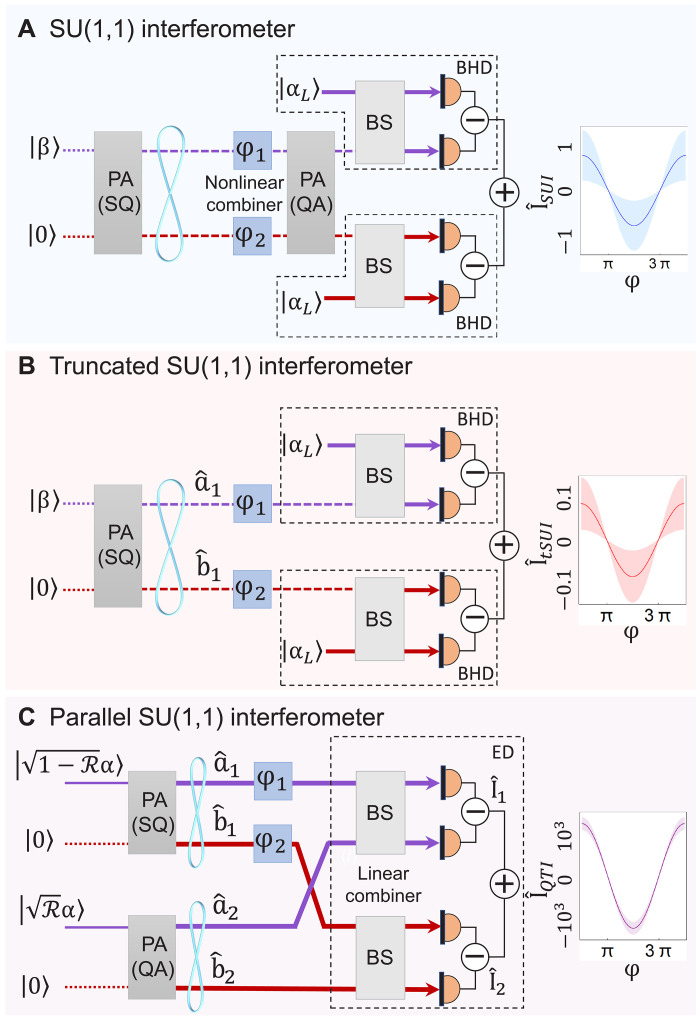
Protocols and schematic diagram of the SUI, tSUI, and QTI. (**A**) The SUI with phase-sensing power $I_{\mathrm{ps}}$ is limited by a nonlinear combiner. (**B**) tSUI with $I_{\mathrm{ps}}\ll \;|\;\alpha_{L}^{2}\;|$ , which is limited by the BHDs. (**C**) pSUI. For R=1∕2 , the fully balanced architecture constitutes the QTI. The right plot shows the output photocurrent for each strategy. Shading indicates the noise fluctuation. $\varphi =\varphi_{1}+\varphi_{2}$ is the total variation of the phase. β , $\sqrt{R}\alpha$ , and $\sqrt{1-R}\alpha$ are amplitudes of seed modes input into each PAs. s is the squeezing parameter for both PAs.

A promising approach for achieving phase measurements beyond the SQL is the SU(1,1) interferometer (SUI) (*15*–*17*), which consists of two cascaded parametric amplification (PA) processes with gain $G_{\mathrm{PA}}$ . The initial PA serves as the squeezer (SQ) generating entangled beams and another PA, actually a quantum amplifier (QA), here functions as a nonlinear combiner for these beams, as illustrated in Fig. 1A. Amplifying slope while maintaining noise levels, the SUI achieves quantum-enhanced phase measurement. The SUI has been successfully demonstrated in various systems, e.g., a full-optical one (*17*–*20*), an atom-light hybrid one (*21*), and an atomic one (*22*–*24*). However, a notable drawback of the SUI lies in its nonlinear combiner, which induces excess losses from mode mismatch and amplifies uncorrelated noises (*25*) while also imposing a restriction of optical power on the phase-sensing light (*26*). This limitation hinders the full exploitation of quantum resources.

One attempt to overcome these limitations has been made by replacing the nonlinear combiner with dual balanced homodyne detection (BHD) in a modified SUI scheme, termed as truncated SUI (tSUI), as shown in Fig. 1B (*27*). However, the restriction on the phase-sensing light power remains unresolved, as a new barrier from BHD is introduced to tSUI. In the BHD, a reference beam with a much stronger power than that of the probe beam is required to amplify the probe beam, avoiding the field fluctuation of the reference beam. In this sense, the BHD actually acts as an imbalanced Mach-Zehnder interferometer (MZI) (*28*) between the reference beam and the probe one with a low fringe visibility due to imbalanced interference. This results in a low utilization efficiency of photon resources in tSUI and poor capability to achieve high photon numbers for phase sensing (see Supplementary Text S1). Consequently, despite its successful implementation in various applications, the sensitivity of the tSUI remains far from satisfactory, as the probe beam’s power has not been fully exploited to its upper limit in these cases (*29*–*33*).

To fully exploit photon resources in such interferometry for phase sensing with high photon number, a new detection configuration is desired to replace the BHD. Here, we present an innovative configuration, “quantum twin interferometer” (QTI), as shown in Fig. 1C, where a second interferometer (colored in red) is cloned from an original one (colored in purple) through the utilization of two PAs. Diverting from the cascaded PAs found in the SUI, we use PAs arranged in parallel as the parallel SU(1,1) interferometer (pSUI), where the optimal working condition of the pSUI corresponds to the QTI. By substituting the nonlinear combiner in the SUI with a linear one, the QTI effectively circumvents the drawbacks associated with the QA, akin to the tSUI.

Breaking through the constraints of both the SUI and tSUI, this comprehensive design enables the QTI to accommodate a substantial amount of phase-sensing power within a balanced interferometer, achieving a large slope while suppressing quantum noises. Experimental results reveal that the QTI reduces quantum noise by 3 dB while increasing the phase-sensing power $I_{\mathrm{ps}}$ by three orders of magnitude compared to previously reported photon-correlated interferometers. Our work on the QTI revolutionizes the optical interferometry with a previously unidentified method of entangled detection (ED), pushing the sensitivity to regimes that previous photon-correlated interferometers struggled to achieve (*17*, *18*, *23*, *27*, *30*, *34*, *35*), thereby opening up prospects for practical applications.

## RESULTS

To highlight the advantages of the QTI, we first compare two detection strategies, BHD and ED. In a general detection framework, a pair of quantum-correlated beams, ${\hat{a}}_{1}$ and ${\hat{b}}_{1}$ , is interfered using another pair of reference beams, ${\hat{a}}_{2}$ and $\;{\hat{b}}_{2}$ . Specifically, ${\hat{a}}_{1}$ and ${\hat{a}}_{2}$ are combined at a beam splitter (BS), and the resulting interference generates the observable ${\hat{I}}_{1}$ . Similarly, ${\hat{b}}_{1}$ and $\;{\hat{b}}_{2}$ are combined at another BS, producing the observable ${\hat{I}}_{2}$ . To determine the optimal sensitivity and track the evolution of the slope and noise, the field operators are decomposed into their mean values and fluctuations as follows

$$\begin{array}{c} {\hat{a}}_{1}=\left\langle {\hat{a}}_{1}\right\rangle +\;\delta {\hat{a}}_{1},{\hat{a}}_{2}=\left\langle {\hat{a}}_{2}\right\rangle +\;\delta {\hat{a}}_{2}\\ {\hat{b}}_{1}=\left\langle {\hat{b}}_{1}\right\rangle +\;\delta {\hat{b}}_{1},{\hat{b}}_{2}=\left\langle {\hat{b}}_{2}\right\rangle +\;\delta {\hat{b}}_{2} \end{array}$$ (2)

At this point, the output observables ${\hat{I}}_{1}$ and ${\hat{I}}_{2}$ can be written as

$$\begin{array}{c} {\hat{I}}_{1}=\;{\hat{a}}_{1}^{†}{\hat{a}}_{2}e^{{i\varphi }_{1}}+\;{\hat{a}}_{2}^{†}{\hat{a}}_{1}e^{-i\varphi_{1}}\\ \;=2|\;\alpha_{1}\;\;||\;\alpha_{2}\;|\text{cos}\varphi_{1}\;+\;\;|\;\alpha_{1}\;|\delta {\hat{X}}_{a_{2}}\left(-\varphi_{1}\right)\;+\;|\;\alpha_{2}\;|\delta {\hat{X}}_{a_{1}}\left(\varphi_{1}\right) \end{array}$$ (3)

$$\begin{array}{c} {\hat{I}}_{2}={\hat{b}}_{1}^{†}{\hat{b}}_{2}e^{{i\varphi }_{2}}+\;{\hat{b}}_{2}^{†}{\hat{b}}_{1}e^{-i\varphi_{2}}\\ \;=2|\;\beta_{1}\;||\;\beta_{2}\;|\text{cos}\varphi_{2}\;+|\;\beta_{1}\;|\delta {\hat{X}}_{b_{2}}\left(-\varphi_{2}\right)\;+|\;\beta_{2}\;|\delta {\hat{X}}_{b_{1}}\left(\varphi_{2}\right) \end{array}$$ (4)

Here, $\delta {\hat{X}}_{k}\left(\gamma \right)=\delta \hat{k}e^{-i\gamma }+\delta {\hat{k}}^{†}e^{i\gamma }$ with $k\in \left\{\;a_{1},a_{2},b_{1},b_{2}\;\right\}$ are the quadratures of mode $\hat{k}$ , and γ is the phase of the quadrature. $\;|\;\alpha_{1}\;|$ , $|\;\alpha_{2}\;|$ , $|\;\beta_{1}\;|$ , and $|\;\beta_{2}\;|$ are the amplitudes of the modes ${\hat{a}}_{1}$ , ${\hat{a}}_{2}$ , ${\hat{b}}_{1}$ , and ${\hat{b}}_{2}$ , respectively. In Eqs. 3 and 4, the first terms stand for the interference signals, whereas the subsequent two terms signify cross-amplified noises. Next, the optical intensities of ${\hat{I}}_{1}$ and ${\hat{I}}_{2}$ can be optimized electronically (*36*), resulting in a total output of

$$\begin{array}{c} \hat{I}=\eta {\hat{I}}_{1}+\;{\hat{I}}_{2}=2I_{\mathrm{ps}}\sqrt{R\left(1-R\right)}\left[\frac{1}{\eta }\text{cos}\varphi_{1}+\text{cos}\varphi_{2}\right]\\ +\sqrt{I_{\mathrm{ps}}}\;\left[\sqrt{1-R}\delta {\hat{X}}_{1}\left(\varphi_{1},\varphi_{2}\right)+\sqrt{R}\delta {\hat{X}}_{2}\left(\varphi_{1},\varphi_{2}\right)\right] \end{array}$$ (5)

Here, the power ratio $R={|\alpha_{1}|}^{2}/\left({|\alpha_{1}|}^{2}+{|\alpha_{2}|}^{2}\right)={|\beta_{1}|}^{2}/\left({|\beta_{1}|}^{2}+{|\beta_{2}|}^{2}\right)$ denotes the ratio of the optical intensity in one arm relative to the combined total intensity of both arms in a single interferometer. $I_{\mathrm{ps}}=\eta^{2}\left({|\;\alpha_{1}\;|}^{2}+{|\;\alpha_{2}\;|}^{2}\right)=\;{|\;\beta_{1}\;|}^{2}+{|\;\beta_{2}\;|}^{2}$ is the total power in the one of the interferometers, where $\eta =|\;\beta_{1}\;|\;/\;|\;\alpha_{1}\;|\;=\;|\;\beta_{2}\;|\;/\;|\;\alpha_{2}\;|$ is the electrical optimal factor. $\delta {\hat{X}}_{1}=\delta {\hat{X}}_{1}\left(\varphi_{1},\varphi_{2}\right)=\delta {\hat{X}}_{a_{1}}\left(\varphi_{1}\right)+\delta {\hat{X}}_{b_{1}}\left(\varphi_{2}\right)$ and $\delta {\hat{X}}_{2}\left(\varphi_{1},\varphi_{2}\right)=\delta {\hat{X}}_{a_{2}}\left(-\varphi_{1}\right)+\;\delta {\hat{X}}_{b_{2}}\left(-\varphi_{2}\right)$ are joint quadratures.

**Fig. 2.**
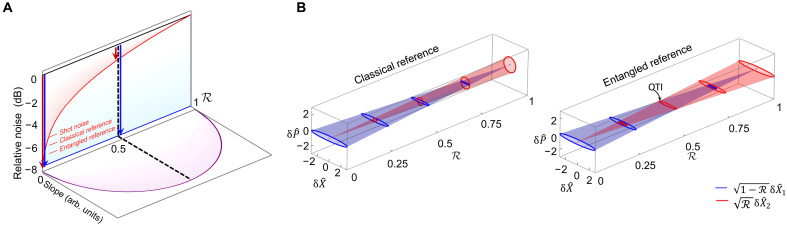
Comparative analysis of signal and noise performance in phase measurement using classical versus entangled reference beams. (**A**) The maximum slope of interference fringe and noise relative to shot noise with the classical (red) and entangled (blue) references varies with the power ratio R. Here, the red and blue arrows denote the quantum enhancements under classical and entangled reference scenarios for R→0 and R = 0.5, respectively. arb. units, arbitrary units. (**B**) Noise contributions of probe ( $\sqrt{1-R}\delta {\hat{X}}_{1}$ ) and reference ( $\sqrt{R}\delta {\hat{X}}_{2}$ ) beams as a function of the power ratio R. When R = 0.5 (black dashed line) approaches the maximum SNR, it corresponds to the condition of the QTI. $\delta \hat{P}$ represents the noise of the phase quadrature, which corresponds to $\varphi_{1}=\varphi_{2}=\pi ∕2$ . $\delta \hat{X}$ represents the noise of the amplitude quadrature, which corresponds to $\varphi_{1}=\varphi_{2}=0$.

Figure 2A presents a comparative plot of relative noise and slope for classical and entangled reference beam configurations as a function of power ratio R. Red and blue traces indicate noise performance with classical and entangled references, respectively. As R approaches 0, the noise contributions from the reference beams ( $\sqrt{R}\delta {\hat{X}}_{2}$ ) become negligible, resulting in equivalent noise reduction for both classical and entangled reference schemes. In this context, the establishment of entanglement between modes ${\hat{a}}_{1}$ and ${\hat{b}}_{1}$ is sufficient to achieve quantum noise reduction in the joint quadrature component $\delta {\hat{X}}_{1}$ . This configuration is formally equivalent to the tSUI, which uses strong references in BHDs. Although the quantum noise can be reduced, the highly imbalanced beam powers lead to poor fringe visibility and thus a significantly weakened signal.

To obtain the maximal signal, the beam powers must be balanced (R →1/2), as illustrated by the purple trace in Fig. 2A. However, noise performance is jointly influenced by probe noise $\sqrt{1-R}\delta {\hat{X}}_{1}$ and reference noise $\sqrt{R}\delta {\hat{X}}_{2}$ under this condition according to Eq. 5. In Fig. 2B, we present a comparative analysis of the state evolution along with the power ratio R in the phase-space ( $\delta \hat{P}-\delta \hat{X}$ ) with measurement strategies for twin beams using classical versus entangled references. The left panel of Fig. 2B presents the traditional strategy using classical reference beams to measure twin beams. As the power ratio R increases, the noise contribution from the entangled probe light $\sqrt{1-R}\delta {\hat{X}}_{1}$ (depicted in blue) gradually diminishes, whereas the noise fraction from the classical reference light $\sqrt{R}\delta {\hat{X}}_{2}\;$ (in red) progressively dominates, driving the system’s noise characteristics to converge toward classical behavior. By contrast, to maintain squeezing across arbitrary power ratio R, reference modes ${\hat{a}}_{2}$ and ${\hat{b}}_{2}$ must be replaced with an entangled beam pair, enabling simultaneous quantum squeezing of the joint quadrature noise $\delta {\hat{X}}_{1}$ and $\delta {\hat{X}}_{2}$ , as the state evolution plotted in the right panel of Fig. 2B, which corresponds to the strategy of the pSUI in Fig. 1C. When the power is equally distributed between the two arms ( R=1∕2 ), the fully symmetric architecture constitutes the QTI, yielding optimal sensitivity.

**Fig. 3.**
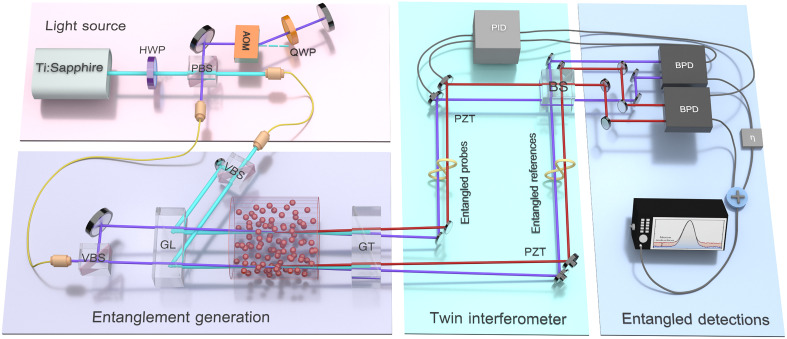
Schematic diagram of the experiment for a twin interferometer. PBS, polarization beam splitter; VBS, variable beam splitter; BS, beam splitter; HWP, half-wave plate; QWP, quarter-wave plate; PZT, piezoelectric transducer; AOM, acousto-optic modulators; GL, Glan-Laser polarizer; GT, Glan-Thompson polarizer; PID, proportion-integration-differentiation; BPD, balanced photodetector; η is the electrical optimal factor.

The experimental arrangement for the QTI is shown in Fig. 3. Two sets of twin beams are generated by two nondegenerate four-wave mixing (FWM) processes with gain $G_{\mathrm{pA}}$ (*37*) (see Supplementary Text S4). The correlated photon with signal and idler frequencies are separately combined at BSs, and the output light is then sent to differential detectors. The dc parts of the subtracted currents are injected into proportion-integration-differentiation (PID) controllers and feedback to the arms of the QTI for locking the phase (*38*). All the phase shifts here are achieved by piezoelectric transducers (PZTs). The ac parts of the differential currents are summed and sent to the spectrum analyzer (SA). The seed power can be adjusted by rotating the half-wave plate before the Glan-Laser polarizer in front of the cells, making it easy to switch between the QTI and another strategy of the pSUI by setting an equal or highly unbalanced seed power for the two FWM processes. It is also flexible to remove the cells, resulting in a switch to the classical MZI achieved by the interference of two seed lights. In the experiment, we set phases $\varphi_{1}=\varphi_{10}+\Delta \varphi_{1}$ and $\varphi_{2}=\varphi_{20}+\Delta \varphi_{2}$ . Here, $\varphi_{10}$ and $\varphi_{20}$ represent the operation points for each interferometer, whereas $\Delta \varphi_{1}$ and $\Delta \varphi_{2}$ are the small signals to be measured. The resulting signal-to-noise ratio (SNR) of the output signal is defined as $\zeta ={\left(\Delta \varphi_{1}+\Delta_{\varphi_{2}}\right)}^{2}∕\delta^{2}\varphi$ . Then, we can acquire the SNR of the pSUI

$$\begin{array}{c} \zeta_{\text{pSUI}}=\frac{4R\left(1-R\right){\left\{\left[\text{cos}h\left(s\right)\Delta \varphi_{1}+\text{sinh}\left(s\right)\Delta \varphi_{2}\right]\text{sinh}\left(s\right){|\;\alpha \;|}^{2}\;\right\}}^{2}}{e^{-2s}{|\;\alpha \;|}^{2}} \end{array}$$ (6)

where s is the squeezing parameter for both PAs. ${|\;\alpha \;|}^{2}$ represents the total intensity of light injected to both PAs.

**Fig. 4.**
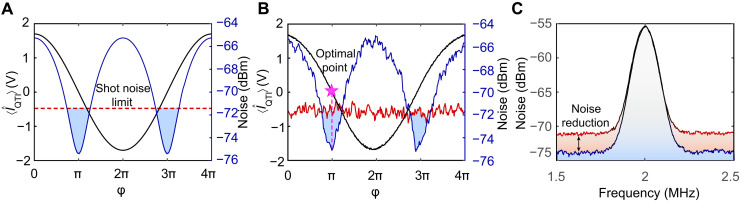
Comparison of spectrum analysis between the QTI and classical MZI performance in phase measurement with the same $I_{\text{ps}}$. (**A**) Theoretical noise power spectra. (**B**) Experimental noise power spectra. Black: signal of the QTI with scanned global operation points ( $\varphi =\varphi_{10}+\varphi_{20}$ ). Blue: noise of the QTI with scanned global phase. Red: MZI locked at minimal noise, indicating shot noise limit. (**C**) SNR comparison between the QTI and MZI with same phase signal at 2 MHz. Black → red: MZI. Black → blue: QTI. Traces are recorded with a 100-kHz resolution bandwidth and 300-Hz video bandwidth. Traces in (B) are averaged three times.

The enhanced performance of the QTI compared to the classical MZI with the same $I_{\mathrm{ps}}$ is illustrated in Fig. 4 (A and B), representing the theoretical and experimental results of their noise power spectra performance, respectively. The red traces indicate the noise level of the MZI with the same $I_{\mathrm{ps}}$ compared to the QTI. The black and blue traces show the signal and noise of the QTI, respectively, when scanning the global operation points ( $\varphi =\varphi_{10}+\varphi_{20}$ ). This reveals a minimal noise level, corresponding to ~3.5-dB suppression in noise compared to the MZI at the same $I_{\mathrm{ps}}$. Figure 4C illustrates the SNR comparison between the QTI and MZI for measuring the phase of $\Delta \varphi_{1}+\Delta \varphi_{2}$ modulated at 2 MHz at the operation point $\varphi_{10}=\varphi_{20}=\pi ∕2$ . The MZI and QTI yield SNR values of 15.5 and 18.5 dB, respectively, showing a 3-dB improvement. Under the same injected light intensity ∣α∣ , R of the tSUI approaches zero. As predicted by Eq. 6, the SNR will be far smaller than those of the QTI and MZI in this scenario, rendering the signal unobservable. The results of the QTI above were obtained with the gain of PAs $G_{\mathrm{PA}}={\text{cosh}}^{2}\left(s\right)=3$ , where both PAs have the same seed power and $I_{\mathrm{ps}}=400\;\mathrm{\mu W}$.

**Fig. 5.**
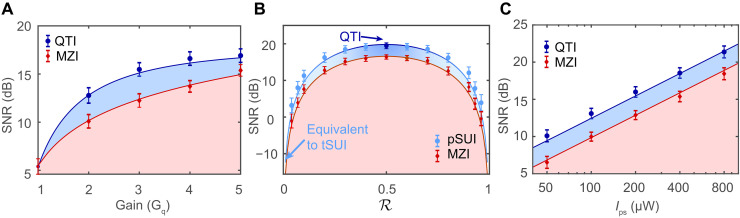
Optimizing the SNR in phase measurement and comparing the performance between the QTI and MZI. Blue: QTI. Red: MZI. (**A**) SNR versus gain $G_{q}$ . (**B**) SNR versus the power ratio R . When R=0.5 , the pSUI becomes the QTI. When R→0 , the pSUI is equivalent to the tSUI. (**C**) SNR versus the intensity of phase-sensing light ( $I_{\mathrm{ps}}$).

To determine the optimal operating conditions, the evolution as a function of various experimental parameters is depicted in Fig. 5. Here, the SNRs of the QTI and MZI are both run at the same $I_{\mathrm{ps}}$ . In Fig. 5A, measurements are taken with R=1/2 and $I_{\mathrm{ps}}=400\;\mathrm{\mu W}$ . The SNR rises with increasing $G_{\mathrm{PA}}$ , reaching saturation around $G_{\mathrm{PA}}\to 5$ . The maximal quantum enhancement occurs when $G_{\mathrm{PA}}={\text{cosh}}^{2}\left(s\right)=3$ , decreasing slightly with each subsequent increase in $G_{\mathrm{PA}}$ . This phenomenon can be described by the imperfect mode matching between spatial multimode twin beams introducing uncorrelated thermal noise (*39*). The SNR versus R is depicted in Fig. 5B. It is easy to observe that quantum enhancement can be maintained with all R. Nevertheless, when the seed power for the two PAs is highly imbalanced, most of the amplified photons go toward the reference beams. In this case, the quantum noise of the reference can be ignored, resulting in the pSUI being equivalent to the tSUI, which exhibits a notably low SNR in such a strategy. Figure 5C illustrates the relationship between the SNR and $I_{\mathrm{ps}}$ , which grows with the increase in seed injection power. The results reveal a proportional growth in SNR with $I_{\mathrm{ps}}$ , and the 3-dB quantum enhancement persists as $I_{\mathrm{ps}}$ approach the order of milliwatts, a magnitude significantly greater than the sub-microwatt levels observed in previous tSUIs (*27*, *35*). A detailed theory (refer to Supplementary Text S3) that takes into account the loss from optical path and modes mismatching fits the experimental data well, as indicated by the solid lines in Fig. 5.

## DISCUSSION

In summary, we use two pairs of twin beams to construct a pair of correlated interferometers as the QTI. We notice that the construction of the QTI is similar to that of the tSUI, both of which have intrinsic mechanisms that build correlations between a pair of interferometers to achieve quantum-enhanced precision measurements. Both the tSUI and QTI feature a facilitated setup compared to the previous quantum interferometer (*17*, *35*) by combining interferometry and signal readout processes, reducing losses such as those from linear and nonlinear mixing. Compared to the weak signal of the tSUI due to the restrictions of classical reference beams, the QTI allows for correlating a pair of interferometers with balanced intensity between the arms, resulting in significantly increased signal strength while preserving all the benefits of the tSUI.

The proposed device exploits positive phase signal correlation and inverse noise correlation at its operating point, allowing for distributed sensing and correlated noise cancellation by summing the outputs of the correlated interferometers. Along with achieving a three-orders-of-magnitude enhancement in $I_{\mathrm{ps}}$ , we observed a 3-dB quantum enhancement. In principle, the sensitivity could approach the Heisenberg scaling by reducing the number of seed photons (see Supplementary Text S2). The robustness of the quantum enhancement across a wide range of $I_{\mathrm{ps}}$ offers flexibility for different types of practical applications, such as biosensing (*31*, *40*) and ultrasensitive measurements of force (*30*, *31*). In contrast to previously reported photon-correlated interferometers (*17*, *27*, *35*), this method does not require additional BHD, thus eliminating the need for extra local oscillator fields and the associated mode-matching issues. This makes the cloning method more practical and readily extendable to other types of optical systems.

In this work, the optical intensity is constrained by technical limitations of acousto-optic modulators (AOMs) and related components, restricting the power of seed light intensity, imposing a corresponding limitation on the $I_{\mathrm{ps}}$ to the milliwatt regime. With the technical limitation imposed by the AOM overcome, the fundamental limit of $I_{\mathrm{ps}}$ remains to be further investigated. In our experiment, quantum light was generated using atomic vapors, making it highly suitable for applications involving interactions with atomic systems. In addition, it is feasible to realize the QTI via nonlinear processes in crystal- or fiber-based systems, thereby expanding its applications to other frequency regimes. As light serves as an excellent information carrier, the measurement of its phase can be converted into determinations of various physical quantities, such as electric fields (*4*), magnetic fields (*41*), and forces (*42*). This conversion capability enables our system to achieve quantum-enhanced precision measurements in a wide range of applications.
