# Supplementary material for: Quantum twin interferometers
Source: Sci Adv. 2025 Oct 31;11(44):eaea0816. doi: 10.1126/sciadv.aea0816 (PMC13142772; doi:10.1126/sciadv.aea0816)
Supplement: Supplementary file 1 — Supplementary Text S1 to S4 Figs. S1 to S3 [file sciadv.aea0816_sm.pdf]

Science Advances
AAAS

Supplementary Materials for

Quantum twin interferometers

Wei Du *et al.*

Corresponding author: Guzhi Bao, guzhibao@sjtu.edu.cn; Weiping Zhang, wpz@sjtu.edu.cn

Sci. Adv. **11**, eaea0816 (2025)
DOI: 10.1126/sciadv.aea0816

This PDF file includes:

Supplementary Text S1 to S4
Figs. S1 to S3

SUPPLEMENTARY MATERIALS

1. THE SNR OF TRUNCATED SU(1,1) INTERFEROMETER

The configuration of truncated SU(1,1) interferometer (tSUI) [27], is shown in Fig.1B. Coherent lights are employed as reference beams to enhance the photon numbers within the interferometer. The intensity of the light field a t the output can be expressed as

$$\hat{I}_{tSUI} = \hat{a}_1^\dagger \hat{a}_{lo} e^{i\varphi_1} + \hat{a}_{lo}^\dagger \hat{a}_1 e^{-i\varphi_1} + \hat{b}_1^\dagger \hat{b}_{lo} e^{i\varphi_2} + \hat{b}_{lo}^\dagger \hat{b}_1 e^{-i\varphi_2} \tag{S1}$$

Where $\hat{a}_{lo}$ and $\hat{b}_{lo}$ denote the modes for the reference light. The optimal sensitivity is achieved when $\varphi_{10} = \pi/2$ and $\varphi_{20} = \pi/2$. The corresponding SNR is

$$\zeta_{tSUI} = \frac{2|\alpha_L|^2|\beta|^2 \left[\cosh(s)\delta\varphi_1 + \sinh(s)\delta\varphi_2\right]^2}{|\alpha_L|^2 e^{-2s} + (|\beta|^2+1)\cosh 2s - 1} \tag{S2}$$

Here s is the squeezing parameter of the PA. We notice that the fluctuations of the classical reference beams will contribute to the noise when the power of the reference beam $|\alpha_L|^2$ is comparable to the power of probe beam $\cosh(2s)|\beta|^2$, resulting in classical features gradually dominating the noise part. The uncorrelated noise can be eliminated, allowing for the effective use of quantum squeezed noise while $|\alpha_L| \gg |\beta|$, leading to $\left\langle \delta^2 \hat{I}_{tSUI} \right\rangle_0 = |\alpha_L|^2 e^{-2s} + (|\beta|^2+1)\cosh(2s) - 1 \approx |\alpha_L|^2 e^{-2s}$. In this scenario, the output photocurrent can be approximated as $I_{tSUI} \propto \sqrt{|\alpha_L|/2}\left[\hat{X}_{\hat{a}_1}(\varphi_1) + \hat{X}_{\hat{b}_1}(\varphi_2)\right]$. This represents a dual homodyne configuration, where the majority of photons serve as the reference beams, with only a small fraction dedicated to measurement. Considering the requirement for highly imbalanced power of probe and reference beams $|\alpha_L| \gg |\beta|$, this approach functions as a combination of two extremely unbalanced interferometers, resulting in a very weak signal. Currently, obtaining a high SNR poses a challenge due to the ineffective utilization of the injected coherent light.

2. THEORETICAL CALCULATION OF QUANTUM TWIN INTERFEROMETER

The quantum optical performance of the parallel arranged SUI (pSUI), termed as Quantum Twin Interferometer (QTI) at the optimal sensitivity, can be analyzed by considering cascaded linear input-output relations, i.e., through a series of linear transformations on field operators.

$$\hat{a}_s = \sqrt{\mathcal{R}}\hat{a}_0 + \sqrt{1-\mathcal{R}}\hat{b}_0 \tag{S3}$$

$$\hat{b}_s = \sqrt{\mathcal{R}}\hat{b}_0 - \sqrt{1-\mathcal{R}}\hat{a}_0 \tag{S4}$$

$$\hat{a}_1 = \cosh(s_1)\hat{a}_s + \sinh(s_1)\hat{a}_i^\dagger \tag{S5}$$

$$\hat{b}_1 = \cosh(s_1)\hat{a}_i + \sinh(s_1)\hat{a}_s^\dagger \tag{S6}$$

$$\hat{a}_2 = \cosh(s_2)\hat{b}_s + \sinh(s_2)\hat{b}_i^\dagger \tag{S7}$$

$$\hat{b}_2 = \cosh(s_2)\hat{b}_i + \sinh(s_2)\hat{b}_s^\dagger \tag{S8}$$

$$\hat{g} = \frac{1}{\sqrt{2}}(e^{i\varphi_1}\hat{a}_1 + \hat{a}_2) \tag{S9}$$

$$\hat{h} = \frac{1}{\sqrt{2}}(e^{i\varphi_1}\hat{a}_1 - \hat{a}_2) \quad \text{(S10)}$$

$$\hat{i} = \frac{1}{\sqrt{2}}(e^{i\varphi_2}\hat{b}_1 + \hat{b}_2) \quad \text{(S11)}$$

$$\hat{j} = \frac{1}{\sqrt{2}}(e^{i\varphi_2}\hat{b}_1 - \hat{b}_2) \quad \text{(S12)}$$

where $s_{1,2}$ symbolizes the squeezing parameter. Here, $\mathcal{R} = |\alpha_1|^2/(|\alpha_1|^2 + |\alpha_2|^2)$ with $|\alpha_1|^2 + |\alpha_2|^2 = |\alpha|^2$ represents the power ratio of one seed beam to the total input beam $\hat{a}_0$ in parallel arranged SUI (pSUI). Ultimately, the output modes are simplified to

$$\hat{g} = \frac{1}{\sqrt{2}}\left\{\left[\left(\cosh(s_1)\sqrt{\mathcal{R}}e^{i\varphi_1} - \cosh(s_2)\sqrt{1-\mathcal{R}}\right)\hat{a}_0 + \left(\cosh(s_1)\sqrt{1-\mathcal{R}}e^{i\varphi_1} + \cosh(s_2)\sqrt{\mathcal{R}}\right)\hat{b}_0\right] + \sinh(s_1)e^{i\varphi_1}\hat{a}_i^\dagger + \sinh(s_2)b_i^\dagger\right\} \quad \text{(S13)}$$

$$\hat{h} = \frac{1}{\sqrt{2}}\left\{\left[\left(\cosh(s_1)\sqrt{\mathcal{R}}e^{i\varphi_1} + \cosh(s_2)\sqrt{1-\mathcal{R}}\right)\hat{a}_0 + \left(\cosh(s_1)\sqrt{1-\mathcal{R}}e^{i\varphi_1} - \cosh(s_2)\sqrt{\mathcal{R}}\right)\hat{b}_0\right] + \sinh(s_1)e^{i\varphi_1}\hat{a}_i^\dagger - \sinh(s_2)b_i^\dagger\right\} \quad \text{(S14)}$$

$$\hat{i} = \frac{1}{\sqrt{2}}\left\{\left[\left(\sinh(s_1)\sqrt{\mathcal{R}}e^{i\varphi_2} - \sinh(s_2)\sqrt{1-\mathcal{R}}\right)\hat{a}_0^\dagger + \left(\sinh(s_1)\sqrt{1-\mathcal{R}}e^{i\varphi_{\varphi_2}} + \sinh(s_2)\sqrt{\mathcal{R}}\right)\hat{b}_0^\dagger\right] + \cosh(s_1)e^{i\varphi_2}\hat{a}_i + \cosh(s_2)b_i\right\} \quad \text{(S15)}$$

$$\hat{j} = \frac{1}{\sqrt{2}}\left\{\left[\left(\sinh(s_1)\sqrt{\mathcal{R}}e^{i\varphi_2} + \sinh(s_2)\sqrt{1-\mathcal{R}}\right)\hat{a}_0^\dagger + \left(\sinh(s_1)\sqrt{1-\mathcal{R}}e^{i\varphi_{\varphi_2}} - \sinh(s_2)\sqrt{\mathcal{R}}\right)\hat{b}_0^\dagger\right] + \cosh(s_1)e^{i\varphi_2}\hat{a}_i - \cosh(s_2)b_i\right\} \quad \text{(S16)}$$

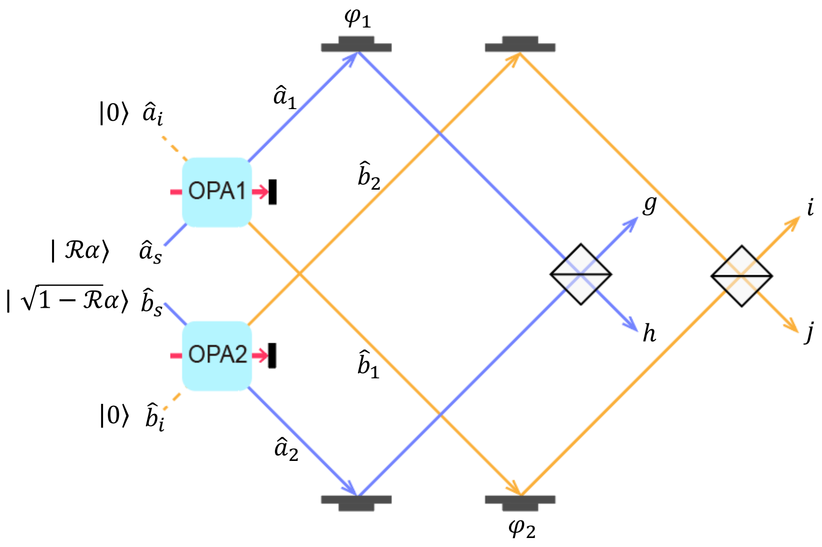

FIG. S1. **Schematic of pSUI**.

The output differential currents in the signal and idler modes become

$$\begin{aligned}\hat{I}_1 &= \hat{g}^\dagger\hat{g} - \hat{h}^\dagger\hat{h} \\ &= -2\cosh(s_1)\cosh(s_2)\sqrt{\mathcal{R}(1-\mathcal{R})}\cos(\varphi_1)\alpha^2 + \cosh(s_1)\cosh(s_2)\left[\mathcal{R}\hat{X}_{b_0}(\varphi_1) - (1-\mathcal{R})\hat{X}_{b_0}(-\varphi_1)\right]\alpha \\ &+ \left[\cosh(s_1)\sinh(s_2)\sqrt{\mathcal{R}}\hat{X}_{bi}(-\varphi_1) - \cosh(s_2)\sinh(s_1)\sqrt{1-\mathcal{R}}\hat{X}_{ai}(\varphi_1)\right]\alpha\end{aligned} \quad \text{(S17)}$$

$$
\begin{aligned}
\hat{I}_2 &= \hat{i}^\dagger \hat{i} - \hat{j}^\dagger \hat{j} \\
&= -2\sinh(s_1)\sinh(s_2)\sqrt{\mathcal{R}(1-\mathcal{R})}\cos(\varphi_2)\alpha^2 + \sinh(s_1)\sinh(s_2)\left[\mathcal{R}\hat{X}_{b_0}(-\varphi_2) - (1-\mathcal{R})\hat{X}_{b_0}(\varphi_2)\right]\alpha \\
&+ \left[\cosh(s_2)\sinh(s_1)\sqrt{\mathcal{R}}\hat{X}_{bi}(\varphi_2) - \cosh(s_1)\sinh(s_2)\sqrt{1-\mathcal{R}}\hat{X}_{ai}(-\varphi_2)\right]\alpha
\end{aligned}
\tag{S18}
$$

Here $\hat{X}_k(\gamma) = \hat{k}e^{-i\gamma} + \hat{k}^\dagger e^{i\gamma}$ with $\beta \in \{b_0, a_i, b_i\}$ are the quadrature of mode $\hat{k}$, and γ is the phase of the quadrature. Then we can get the sum of the differential currents

$$
\begin{aligned}
\hat{I} &= \hat{I}_1 + \hat{I}_2 \\
&= -2\sqrt{\mathcal{R}(1-\mathcal{R})}\left[\cosh(s_1)\cosh(s_2)\cos(\varphi_1) + \sinh(s_1)\sinh(s_2)\cos(\varphi_2)\right]\alpha^2 \\
&+ \mathcal{R}\left[\cosh(s_1)\cosh(s_2)\hat{X}_{b_0}(\varphi_1) + \sinh(s_1)\sinh(s_2)\hat{X}_{b_0}(-\varphi_2)\right]\alpha \\
&- (1-\mathcal{R})\left[\cosh(s_1)\cosh(s_2)\hat{X}_{b_0}(-\varphi_1) + \sinh(s_1)\sinh(s_2)\hat{X}_{b_0}(\varphi_2)\right]\alpha \\
&+ \sqrt{\mathcal{R}}\left[\cosh(s_1)\sinh(s_2)\hat{X}_{b_i}(-\varphi_1) + \cosh(s_2)\sinh(s_1)\hat{X}_{b_i}(\varphi_2)\right]\alpha \\
&- \sqrt{1-\mathcal{R}}\left[\cosh(s_1)\sinh(s_2)\hat{X}_{a_i}(-\varphi_2) + \cosh(s_2)\sinh(s_1)\hat{X}_{a_i}(\varphi_1)\right]\alpha
\end{aligned}
\tag{S19}
$$

The optimal SNR is achieved when $\varphi_1 = \varphi_{10} + \Delta\varphi_1$ and $\varphi_2 = \varphi_{20} + \Delta\varphi_2$ with the operating point $\varphi_{10} = \pi/2$, $\varphi_{20} = \pi/2$, the small signal to be measured $\Delta\varphi_1 \to 0$ and $\Delta\varphi_2 \to 0$, allowing us to acquire the maximum signal and minimal noise simultaneously

$$
\begin{aligned}
\left\langle \Delta\hat{I} \right\rangle^2 &= (\left\langle \hat{I} \right\rangle - \left\langle \hat{I} \right\rangle_0)^2 \\
&= \left\{2\sqrt{\mathcal{R}(1-\mathcal{R})}\left[\cosh(s_1)\cosh(s_2)\Delta\varphi_1 + \sinh(s_1)\sinh(s_2)\Delta\varphi_2\right]\alpha^2\right\}^2
\end{aligned}
\tag{S20}
$$

$$
\left\langle \delta^2\hat{I} \right\rangle_0 = \left\{\left[\cosh(s_1)\cosh(s_2) - \sinh(s_1)\sinh(s_2)\right] + \left[\cosh(s_2)\sinh(s_1) - \cosh(s_1)\sinh(s_2)\right]\right\}\alpha^2 \tag{S21}
$$

Here $\langle I \rangle_0$ represents the expected output with $\varphi_1 = \varphi_{10}$ and $\varphi_2 = \varphi_{20}$, denoting an undisturbed interferometer. $\langle I \rangle$ denotes the expectation with $\varphi_1 = \varphi_{10} + \Delta\varphi_1$ and $\varphi_2 = \varphi_{10} + \Delta\varphi_2$, in the presence of weak signals causing slight phase excursion in the interferometer. Finally, we get the SNR for the pSUI

$$
\zeta_{pSUI} = \frac{\left\{2\sqrt{\mathcal{R}(1-\mathcal{R})}\left[\cosh(s_1)\cosh(s_2)\Delta\varphi_1 + \sinh(s_1)\sinh(s_2)\Delta\varphi_2\right]\alpha^2\right\}^2}{\left\{\left[\cosh(s_1)\cosh(s_2) - \sinh(s_1)\sinh(s_2)\right] + \left[\cosh(s_2)\sinh(s_1) - \cosh(s_1)\sinh(s_2)\right]\right\}\alpha^2} \tag{S22}
$$

The SNR reaches maximum when $\mathcal{R} = 1/2$, which is equal to the SNR of dual-beam SU(1,1) interferometer (SUI) [34]. In this case, we call it QTI. When the signals are induced with common-mode phase $\Delta\varphi_1 = \Delta\varphi_2 \to \Delta\varphi_{sig}$ and $s_1 = s_2 = s$, we get the minimum sensitivity in phase measurement

$$
\delta\varphi_m = \frac{1}{\sqrt{\cosh(2s)I_{ps}}} \tag{S23}
$$

Here $I_{ps} = \left[\cosh^2(s) + \sinh^2(s)\right]\alpha^2 = \cosh(2s)\alpha^2$ is the power of the phase sensing field. When $\alpha \to 1$, we find the sensitivity approach the Heisenberg limit

$$
\delta\varphi_{HL} = \frac{1}{I_{ps}} \tag{S24}
$$

The difference between tSUI and QTI primarily arises from different detection strategies. Here, we directly compare the results from measuring entangled probe beams with balanced homodyne detection (BHD) and entangled detection (ED). Unlike the description in the main text, where the total optical intensity is kept constant, here we fix the intensity of the entangled probe beam while varying the intensity of the reference beam in the two detection schemes. The power ratio $\mathcal{R}$, which represents the power of entangled probe beam relative to the total interference power. For EDs, $\mathcal{R} = |\alpha_1|^2/(|\alpha_1|^2 + |\alpha_2|^2)$. For HD, $\mathcal{R} = |\cosh(2s)\beta|^2/(\cosh(2s)|\beta|^2 + |\alpha_L|^2)$. The signals for both HD and ED exhibit the same dependence on $\mathcal{R}$. However, in HD, where coherent light is used as the reference, the quantum advantage gradually diminishes with the increase of $\mathcal{R}$. In contrast, in EDs, since the reference is also entangled, the noise remains independent of $\mathcal{R}$.

3. QUANTUM TWIN INTERFEROMETER WITH LOSSES

In practical experiments, losses are unavoidable. Here, we discuss the losses caused by optical path and mode mismatch in interferometry. In such cases, the input-output relation changes to

$$\hat{a}_1 = \sqrt{1-\kappa_s}\left[\cosh(s)\hat{a}_s + \sinh(s)\hat{a}_i^\dagger\right] + \sqrt{\kappa_s}\hat{L}_{sv} \tag{S25}$$

$$\hat{b}_1 = \sqrt{1-\kappa_i}\left[\cosh(s)\hat{a}_i + \sinh(s)\hat{a}_s^\dagger\right] + \sqrt{\kappa_i}\hat{L}_{iv} \tag{S26}$$

$$\hat{a}_2 = \sqrt{1-\kappa_s}\left[\cosh(s)\hat{b}_s + \sinh(s)\hat{b}_i^\dagger\right] + \sqrt{\kappa_s}\hat{L}_{sv} \tag{S27}$$

$$\hat{b}_2 = \sqrt{1-\kappa_i}\left[\cosh(s)\hat{b}_i + \sinh(s)\hat{b}_s^\dagger\right] + \sqrt{\kappa_i}\hat{L}_{iv} \tag{S28}$$

$$\hat{g} = \frac{1}{\sqrt{2}}\sqrt{1-\sigma_s}(e^{i\varphi_1}\hat{a}_1 + \hat{a}_2) + \sqrt{\sigma_s}\hat{L}_{st} \tag{S29}$$

$$\hat{h} = \frac{1}{\sqrt{2}}\sqrt{1-\sigma_s}(e^{i\varphi_1}\hat{a}_1 - \hat{a}_2) + \sqrt{\sigma_s}\hat{L}_{st} \tag{S30}$$

$$\hat{i} = \frac{1}{\sqrt{2}}\sqrt{1-\sigma_i}(e^{i\varphi_2}\hat{b}_1 + \hat{b}_2) + \sqrt{\sigma_i}\hat{L}_{it} \tag{S31}$$

$$\hat{j} = \frac{1}{\sqrt{2}}\sqrt{1-\sigma_i}(e^{i\varphi_2}\hat{b}_1 - \hat{b}_2) + \sqrt{\sigma_i}\hat{L}_{it} \tag{S32}$$

Here κ_s and κ_i represent losses from the optical path, while σ_s and σ_i represent losses from mode mismatch as illustrated in Fig. S2. $\hat{L}_t$ with $t \in \{sv, iv\}$ are the vacuum noise induced from the optical path loss and $t \in \{st, it\}$ are the thermal noise induced from the mode mismatch. The output modes are simplified to

$$\begin{aligned}\hat{g} = &\frac{\sqrt{(1-\kappa_s)(1-\sigma_s)}}{\sqrt{2}}\left\{\cosh(s)\left[\left(\sqrt{\mathcal{R}}e^{i\varphi_1} - \sqrt{1-\mathcal{R}}\right)\hat{a}_0 + \left(\sqrt{1-\mathcal{R}}e^{i\varphi_1} + \sqrt{\mathcal{R}}\right)\hat{b}_0\right] + \sinh(s)\left(e^{i\varphi_1}\hat{a}_i^\dagger + b_i^\dagger\right)\right\} \\ &+ \frac{\sqrt{\kappa_s(1-\sigma_s)}}{\sqrt{2}}(1+e^{i\varphi_1})\hat{L}_{sv} + \sqrt{\sigma_s}\hat{L}_{st}\end{aligned} \tag{S33}$$

$$\begin{aligned}\hat{h} = &\frac{\sqrt{(1-\kappa_s)(1-\sigma_s)}}{\sqrt{2}}\left\{\cosh(s)\left[\left(\sqrt{\mathcal{R}}e^{i\varphi_1} - \sqrt{1-\mathcal{R}}\right)\hat{a}_0 + \left(\sqrt{1-\mathcal{R}}e^{i\varphi_1} + \sqrt{\mathcal{R}}\right)\hat{b}_0\right] + \sinh(s)\left(e^{i\varphi_1}\hat{a}_i^\dagger + b_i^\dagger\right)\right\} \\ &+ \frac{\sqrt{\kappa_s(1-\sigma_s)}}{\sqrt{2}}(e^{i\varphi_1}-1)\hat{L}_{sv} + \sqrt{\sigma_s}\hat{L}_{st}\end{aligned} \tag{S34}$$

$$\begin{aligned}\hat{i} = &\frac{\sqrt{(1-\kappa_i)(1-\sigma_i)}}{\sqrt{2}}\left\{\sinh(s)\left[\left(\sqrt{\mathcal{R}}e^{i\varphi_2} - \sqrt{1-\mathcal{R}}\right)\hat{a}_0^\dagger + \left(\sqrt{1-\mathcal{R}}e^{i\varphi_{\varphi_2}} + \sqrt{\mathcal{R}}\right)\hat{b}_0^\dagger\right] + \cosh(s)\left(e^{i\varphi_2}\hat{a}_i + b_i\right)\right\} \\ &+ \frac{\sqrt{\kappa_i(1-\sigma_i)}}{\sqrt{2}}(1+e^{i\varphi_2})\hat{L}_{iv} + \sqrt{\sigma_i}\hat{L}_{it}\end{aligned} \tag{S35}$$

$$\begin{aligned}\hat{j} = &\frac{\sqrt{(1-\kappa_i)(1-\sigma_i)}}{\sqrt{2}}\left\{\sinh(s)\left[\left(\sqrt{\mathcal{R}}e^{i\varphi_2} + \sqrt{1-\mathcal{R}}\right)\hat{a}_0^\dagger + \left(\sqrt{1-\mathcal{R}}e^{i\varphi_2} - \sqrt{\mathcal{R}}\right)\hat{b}_0^\dagger\right] + \cosh(s)\left(e^{i\varphi_2}\hat{a}_i - b_i\right)\right\} \\ &+ \frac{\sqrt{\kappa_i(1-\sigma_i)}}{\sqrt{2}}(e^{i\varphi_2}-1)\hat{L}_{iv} + \sqrt{\sigma_i}\hat{L}_{it}\end{aligned} \tag{S36}$$

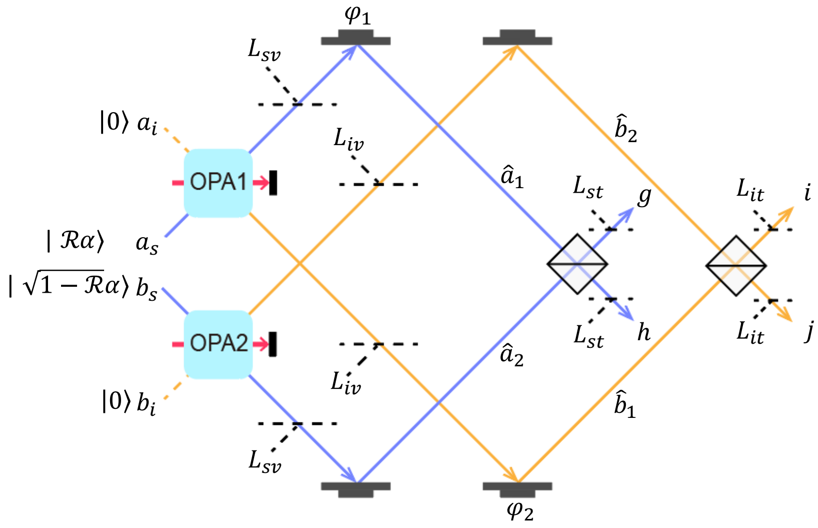

FIG. S2. **Twin Interferometer when losses are considered.**

The output differential current in signal and idler modes become

$$\begin{aligned}\hat{I}_1 &= \hat{g}^\dagger\hat{g} - \hat{h}^\dagger\hat{h}\\ &= (1-\kappa_s)(1-\sigma_s)\left\{-2\cosh^2(s)\sqrt{\mathcal{R}(1-\mathcal{R})}\cos(\varphi_1)\alpha^2 + \cosh^2(s)\left[\mathcal{R}\hat{X}_{b_0}(\varphi_1) - (1-\mathcal{R})\hat{X}_{b_0}(-\varphi_1)\right]\alpha\right.\\ &\left.+\cosh(s)\sinh(s)\left[\sqrt{\mathcal{R}}\hat{X}_{bi}(-\varphi_1) - \sqrt{1-\mathcal{R}}\hat{X}_{ai}(\varphi_1)\right]\alpha\right\} - (1-\sigma_s)\sqrt{(1-\mathcal{R})\kappa_s(1-\kappa_s)}\cosh(s)\hat{X}_{Lsv}(-\varphi_1)\alpha\\ &- \sqrt{2R\sigma_s(1-\kappa_s)(1-\sigma_s)}\cosh(s)\hat{X}_{Lst}(0)\alpha\end{aligned} \tag{S37}$$

$$\begin{aligned}\hat{I}_2 &= \hat{i}^\dagger\hat{i} - \hat{j}^\dagger\hat{j}\\ &= (1-\kappa_i)(1-\sigma_i)\left\{-2\sinh^2(s)\sqrt{\mathcal{R}(1-\mathcal{R})}\cos(\varphi_2)\alpha^2 + \sinh^2(s)\left[\mathcal{R}\hat{X}_{b_0}(-\varphi_2) - (1-\mathcal{R})\hat{X}_{b_0}(\varphi_2)\right]\alpha\right.\\ &\left.+\cosh(s)\sinh(s)\left[\sqrt{\mathcal{R}}\hat{X}_{bi}(\varphi_2) - \sqrt{1-\mathcal{R}}\hat{X}_{ai}(-\varphi_2)\right]\alpha\right\} - (1-\sigma_i)\sqrt{(1-\mathcal{R})\kappa_i(1-\kappa_i)}\sinh(s)\hat{X}_{Liv}(-\varphi_2)\alpha\\ &- \sqrt{2R\sigma_i(1-\kappa_i)(1-\sigma_i)}\sinh(s)\hat{X}_{Lit}(0)\alpha\end{aligned} \tag{S38}$$

The optimal SNR can be acquired when $\mathcal{R} = 1/2$, $\varphi_1 \to \varphi_{10} + \Delta\varphi_1$ and $\varphi_2 \to \varphi_{20} + \Delta\varphi_2$ with the operating point $\varphi_{10} = \varphi_{20} = \pi/2$, and weak signal $\Delta\varphi_1 \to 0$, $\Delta\varphi_2 \to 0$. There might be uneven losses among the interferometers in signal and idler modes due to different optical paths, leading to varying intensity and fluctuations between the currents I_1 and I_2. To address this issure, an electrical attenuation with factor $\eta = [\sinh(s)(1-\sigma_i)(1-\kappa_i)]/[\cosh(s)(1-\sigma_s)(1-\kappa_s)]$ is introduced to equalize the losses, yielding the subsequent output

$$\begin{aligned}\hat{I} &= \eta\hat{I}_1 + \hat{I}_2\\ &= (1-\kappa_i)(1-\sigma_i)\sinh(s)\alpha\left\{[\cosh(s)\Delta\varphi_1 + \sinh(s)\Delta\varphi_2]\alpha + e^{-s}\hat{X}_{b_0}(\pi/2)\right\}\\ &- \frac{(1-\sigma_i)(1-\kappa_i)}{(1-\sigma_s)(1-\kappa_s)}\sinh(s)\alpha\left[(1-\sigma_s)\sqrt{\frac{\kappa_s(1-\kappa_s)}{2}}\hat{X}_{Lsv}(-\pi/2) + \sqrt{\sigma_s(1-\kappa_s)(1-\sigma_s)}\hat{X}_{Lst}(0)\right]\\ &- \sinh(s)\alpha\left[(1-\sigma_i)\sqrt{\frac{\kappa_i(1-\kappa_i)}{2}}\hat{X}_{Liv}(-\pi/2) + \sqrt{\sigma_i(1-\kappa_i)(1-\sigma_i)}\hat{X}_{Lit}(0)\right]\end{aligned} \tag{S39}$$

We get the signal

$$\langle\Delta I\rangle^2 = \sinh^2(s)\alpha^2\left\{(1-\kappa_i)(1-\sigma_i)[\cosh(s)\Delta\varphi_1 + \sinh(s)\Delta\varphi_2]\alpha\right\}^2 \tag{S40}$$

Here, the modes $\hat{L}_{sv}$ and $\hat{L}_{iv}$ represent vacuum states, while the modes $\hat{L}_{st}$ and $\hat{L}_{it}$ correspond to thermal states arising from mode-mismatch in interferometry. As a result, the noise levels are $\left\langle \delta^2 \hat{X}_{Lsv} \right\rangle = 1$ and $\left\langle \delta^2 \hat{X}_{Liv} \right\rangle = 1$. Additionally, $\left\langle \delta^2 \hat{X}_{Lst} \right\rangle = e^{2s}$ and $\left\langle \delta^2 \hat{X}_{Lit} \right\rangle = e^{2s}$, with their fluctuations depending on the gain of the PA processes. This leads to the determination of the noise performance of QTI at the output

$$\left\langle \delta^2 \hat{I} \right\rangle = \sinh^2(s)\alpha^2 \left\{ \left[e^{-s}(1-\kappa_i)(1-\sigma_i)\right]^2 + \left[\frac{(1-\sigma_i)(1-\kappa_i)}{(1-\sigma_s)(1-\kappa_s)}\right]^2 \left[\frac{\kappa_s(1-\kappa_s)(1-\sigma_s)^2}{2} + e^{2s}\sigma_s(1-\kappa_s)(1-\sigma_s)\right] + \left[\frac{\kappa_i(1-\kappa_i)(1-\sigma_i)^2}{2} + e^{2s}\sigma_i(1-\kappa_i)(1-\sigma_i)\right] \right\} \tag{S41}$$

Finally, we get the SNR

$$\zeta_{QTI} = \frac{\left\{(1-\kappa_i)(1-\sigma_i)\left[\cosh(s)\Delta\varphi_1 + \sinh(s)\Delta\varphi_2\right]\alpha\right\}^2}{\left\{ \left[e^{-s}(1-\kappa_i)(1-\sigma_i)\right]^2 + \left[\frac{(1-\sigma_i)(1-\kappa_i)}{(1-\sigma_s)(1-\kappa_s)}\right]^2 \left[\frac{\kappa_s(1-\kappa_s)(1-\sigma_s)^2}{2} + e^{2s}\sigma_s(1-\kappa_s)(1-\sigma_s)\right] + \left[\frac{\kappa_i(1-\kappa_i)(1-\sigma_i)^2}{2} + e^{2s}\sigma_i(1-\kappa_i)(1-\sigma_i)\right] \right\}} \tag{S42}$$

For QTI, the power of phase sensing light is $I_{QTI} = \cosh(2s)\alpha^2/2$. Our system can be easily converted to the conventional Mach-Zehnder interferometer (MZI) by setting $s = 0$. For a fair comparison, the weak signal in MZI should satisfy $\delta\varphi_{mz} = \Delta\varphi_1 + \Delta\varphi_2$, and from which we obtain the SNR for MZI

$$\zeta_{MZI} = \frac{\left[(1-\kappa_s)(1-\sigma_s)\Delta\varphi_{mz}\alpha\right]^2}{\left[(1-\kappa_s)(1-\sigma_s)\right]^2 + \left[\frac{\kappa_s(1-\kappa_s)(1-\sigma_s)^2}{2} + \sigma_s(1-\kappa_s)(1-\sigma_s)\right]} \tag{S43}$$

Here, the power of phase sensing light for MZI is $I_{MZI} = \alpha^2/2$. Eq. S42 and Eq. S43 are used in fitting t o the measured data, noting that all traces in Fig. 4 meet the condition $I_{QTI} = I_{MZI}$ for a fair comparison. We find that the theory aligns well with the experimental results. The optimal fitting parameters are $\kappa_s = 0.2$, $\kappa_i = 0.1$, $\sigma_s = 0.03$, and $\sigma_i = 0.02$.

4. GENERATION OF TWIN BEAMS

In our system, the PAs are achieved by the four-wave-mixing (FWM) process for generating the twin beams, whose interaction Hamiltonian is

$$\hat{H}_n = i\hbar\xi \hat{a}_n^\dagger \hat{b}_n^\dagger + h.c \tag{S44}$$

where $n \in (1,2)$ represents the photons generated from SQ or QA. ξ is the strength of interaction which depends on pump power, one-photon detuning, two-photon detuning, etc.

In this experiment, the pump beam is supplied by a Ti: sapphire laser, whose frequency is locked at blue-shifted by approximately $\Delta = 1\,\mathrm{GHz}$ above the transition line of the D1 line of ^{85}Rb $5\,S_{1/2} \to 5\,P_{1/2}$, 795 nm. The seed light is red-shifted by 3.38 GHz from the pump beams through the double pass configuration of a 1.5 GHz acoustic-optic modulator (AOM). The weak seed beam with a waist of 250 μm intersects a strong pump beam with a waist of 500 μm at an angle of 0.3° in a 12 mm long ^{85}Rb vapor cell maintained at a temperature of 120 °C. Two pairs of correlated photons are generated after the FWM processes. The correlated photons are normally referred to as 'signal' and 'idler' with approximately a 6 GHz frequency difference, denoted by modes $\hat{a}_n$ and $\hat{b}_n$ with $n \in (1,2)$ representing photons from the first or second FWM processes as outlined in Eq. S44. Energy level diagram in the D_1 line of ^{85}Rb for FWM process is given in the Fig. S3. Here Δ is the one-photon detuning, and δ is two-photon detuning. P_1 denotes the FWM pump field.

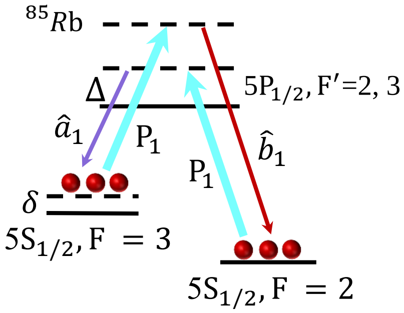

FIG. S3. **Energy level diagram of ^{85}Rb.**
